# Knowledge, beliefs, and attitudes of the Quebec population toward chronic pain: Where are we now?

**DOI:** 10.1080/24740527.2017.1369849

**Published:** 2017-10-24

**Authors:** Anaïs Lacasse, Manon Choinière, Judy-Ann Connelly

**Affiliations:** a Département des sciences de la santé, Université du Québec en Abitibi-Témiscamingue, Rouyn-Noranda, QC, Canada; b Quebec Pain Research Network (QPRN), Université de Sherbrooke, Sherbrooke, QC, Canada; c Centre de recherche du Centre hospitalier de l’Université de Montréal (CRCHUM), Tour Saint-Antoine, Montréal, QC, Canada; d Département d’anesthésiologie, Faculté de médecine, Université de Montréal, Montreal, QC, Canada

**Keywords:** chronic pain, knowledge, beliefs, attitudes, stigmatization, general population, general public, web-based survey

## Abstract

**Background**: Many chronic pain (CP) awareness and educational initiatives have been achieved, but it is time to take stock of where we are today.

**Aims**: The aim of this study was to describe and identify determinants of knowledge, beliefs, and attitudes of different subgroups of the Quebec population regarding CP and especially toward people suffering from this condition.

**Methods**: A web-based, cross-sectional study was conducted between May and June 2014.

**Results**: A total of 1958 participants responded, among whom 70.9% reported suffering from CP and 14.4% reported being a health care professional (HCP). Almost half of the participants were not aware that the risk of developing CP is increased after undergoing surgery or that CP affects approximately one in five adults. A minority (10.30%) agreed that HCP are well trained in CP treatment. The two most frequent negative beliefs were that people suffering from CP become dependent on their medications as do drug addicts (16.7%) and that consulting a psychologist is useless unless the person with CP is depressed (16.9%). Multiple regression analysis showed that being a woman, being born in Canada, being unemployed, suffering from CP, and being an HCP were predictors of better knowledge and more positive attitudes toward people suffering from CP (all *P* values < 0.05). Older age and residing in a remote region were associated with poorer knowledge and more negative attitudes.

**Conclusions**: Our results underline the importance of continuing the efforts and the need for more education programs, awareness campaigns, and stigma reduction activities about CP for HCP, patients, and the general public.

## RÉSUMÉ

**Contexte** : De nombreuses initiatives de sensibilisation et d’éducation à propos de la douleur chronique ont été réalisées mais le temps est venu de faire le point sur la situation actuelle. **Objectifs **: Décrire et identifier les déterminants des connaissances, des attitudes et des croyances de différents sous-groupes de la population du Québec en ce qui concerne la douleur chronique, en particulier à l’égard des personnes qui souffrent de cette condition. **Méthodes **: Une étude transversale en ligne a été menée entre mai et juin 2014. **Résultats **: Au total, 1 958 participants ont répondu, parmi lesquels 70,9 % ont déclaré souffrir de douleur chronique et 14,4 % ont déclaré être des professionnels de la santé. Près de la moitié des participants ne savaient pas que le risque de souffrir de douleur chronique augmente après une intervention chirurgicale ou que la douleur chronique affecte près d’un adulte sur cinq. Une minorité (10,30 %) était d’accord pour dire que les professionnels de la santé sont bien formés pour traiter la douleur chronique. Les deux croyances négatives les plus fréquentes étaient que les personnes souffrant de douleur chronique deviennent dépendantes à leur médication tout comme des toxicomanes (16,7 %) et qu’il est inutile de consulter un psychologue sauf si la personne souffrant de douleur chronique est déprimée (16,9 %). Une analyse de régression multiple a démontré que le fait d’être une femme, d’être né au Canada, d’être sans emploi, de souffrir de douleur chronique et d’être un professionnel de la santé étaient des prédicteurs d’une meilleure connaissance ou d’attitudes plus positives à l’égard des personnes souffrant de douleur chronique (toutes les valeurs p < 0,05). Le fait d’être plus âgé et de vivre dans une région éloignée était associé à des connaissances moindres et à davantage d’attitudes négatives. **Conclusions **: Nos résultats démontrent l’importance de poursuivre les efforts et qu’il est nécessaire de mettre en œuvre davantage de programmes éducatifs, de campagnes de sensibilisation et d’activités visant à réduire la stigmatisation liée à la douleur chronique destinés aux professionnels de la santé, aux patients et au grand public.

## Introduction

Often defined as pain that persists for more than 3–6 months,^1,2^ chronic pain (CP) affects approximately 20% of the adult population worldwide.^3^ This condition impacts all spheres of everyday life of those suffering from it^3,4^ and constitutes a significant economic burden to society.^5,6^ Nevertheless, CP is still underreported, underdiagnosed, and underrecognized in clinical practice and among the general public.^7–12^ Despite decades of research in the field, CP management continues to be far from optimal, with patients going from one doctor to another and seeking different health care treatments^13,14^ Various factors can explain this situation: (1) knowledge gaps and lack of training of health care professionals, (2) limited access to pain clinics and resources, (3) suboptimal use or efficacy of treatment modalities, and (4) lack of recognition of this condition, particularly in primary care settings.^10–12,15–17^ Stigmatization and lack of empathy toward CP sufferers from people of the community, including health care professionals, is also reported.^18–20^ Greater awareness and better education regarding CP for health care professionals, patients, decision makers, and the general public are thus warranted and should be prioritized to improve CP management.^11,12,15,21^

Many studies on the knowledge, beliefs, and attitudes toward CP have been conducted in Canada among health care professionals (e.g., nurses, physicians, pharmacists, physiotherapists, etc.)^2,22–31^ or patients.^32–36^ Understanding the beliefs and attitudes of the general population as a whole is also important and can permit better designing and tailoring of education programs, awareness campaigns and stigma reduction activities. As of now, a substantial number of studies of the general public have been conducted in Canada^37^ and elsewhere^38–41^ regarding perceptions toward back pain or opioids.^42,43^ However, little recent empirical work has been conducted to better understand beliefs and attitudes of the population toward people suffering from CP, regardless of the type of CP they are suffering from or the treatment they are using. Research about determinants of knowledge gaps and negative attitudes toward people suffering from CP is also lacking. In recent years, many CP awareness and educational initiatives have been developed and implemented in Canada,^44–48^ but it is time to take stock of where we are.

The objectives of this study were to (1) describe the knowledge, beliefs, and attitudes of different subgroups of the Quebec population regarding CP, and especially toward people suffering from this condition, and (2) identify participants’ characteristics associated with these perceptions.

## Methods

### Design and settings

A web-based, cross-sectional study was conducted in the population of the province of Quebec between May and June 2014. Residents of this east-central province of Canada, divided into 17 administrative regions, are predominantly French-speaking (85.5%).^49^ Residents whose mother tongue was French and who were at least 18 years of age were eligible to participate in this study. Approval was obtained from the Institutional Ethical Review Board of the Université du Québec en Abitibi-Témiscamingue and all participants provided informed consent to participate.

### Procedure

The complete strategy of our province-wide recruitment procedures has been described elsewhere in the context of a study aimed at developing a French-Canadian scale that could be used to measure knowledge, beliefs, and attitudes that people in the community have toward CP.^50^ To maximize the study sample diversity and representativeness, the invitation to complete the web-based questionnaire was made available on diverse diffusion platforms: (1) organizations that were willing to freely advertise the study invitation on their website, through their member mailing lists, or during their scheduled activities; (2) social media such as Facebook and Twitter; (3) provincial-wide mass media through paid publicity (ads in e-papers and newspapers); and (4) e-mails the study team sent to Quebec provincial research networks, colleagues, and contacts. The questionnaire was available for 6 weeks (May 1 to June 11, 2014).

### Measures

The web-based questionnaire included items to capture knowledge and beliefs about specific aspects of the definition of CP, frequency, and risk factors (true/false/don’t know questions). Participants’ opinions about health care professionals’ training in CP management was measured using a 5-point Likert scale (respondents were asked to report their level of agreement with the following statement: “Health care professionals such as physicians, pharmacists and nurses are well trained in CP management”).

The questionnaire also included the Chronic Pain Myth Scale (CPMS),^50^ a self-administered instrument to measure knowledge, beliefs, and attitudes of the general public toward people suffering from CP (subscale 1), biopsychosocial impacts of CP (subscale 2), and treatment of CP (subscale 3). The CPMS includes 26 items each answered on 5-point Likert scales ranging from 1 (*completely disagree*) to 5 (*completely agree*). Higher values on positively worded items indicate higher knowledge and more positive beliefs and attitudes. Scores on negatively worded items are reversed so that a high value also indicates better perceptions. The score of the three subscales of the CPMS can be obtained by summing items within each dimension, the first subscale being the primary interest of the present study. Preliminary insights into the psychometric qualities of the CPMS in terms of internal structure, internal consistency, and construct validity were provided in the first phase of the present study (sample composed of French-speaking individuals of the province of Quebec and weighted toward women with chronic pain).^50^

Numerous questions were used to establish the socioeconomic profile of participants, such as age, sex, country of birth, employment status, living conditions, annual family income, educational level, and region of residency. Participants were also asked whether they had experienced pain for ≥3 months (considered as CP), knew someone suffering from pain for ≥3 months, had worked or studied in the field of health, had worked with patients claiming CP disability benefits, or were health care professionals (i.e., physicians, nurses, physiotherapists, psychologists, or pharmacists). Although many other types of health care professionals can be involved in the management of CP patients, professionals were chosen because the first four were identified by the International Association for the Study of Pain as clinicians who should ideally be involved in multidisciplinary pain management.^51^ Pharmacists were added because they were reported to be the second most commonly used source of information for pain management after general practitioners.^52^ At the very end of the web-based questionnaire, some key points were presented to study participants for their personal benefit and as a means of increasing awareness and education (e.g., definition of CP, its prevalence, and its impacts). After reading this information, respondents could not go back to edit previous pages of the questionnaire.

Before the beginning of the study, the questionnaire was reviewed by pain experts for content coverage, relevance, and clarity and pretested in individuals from the general population from various socioeconomic statuses to ensure adequate comprehension of the items.

### Statistical analysis

Descriptive statistics—that is, means, standard deviations (SDs), and frequency tables (*n* and %)—were calculated to summarize participants’ characteristics and knowledge and beliefs about specific aspects of CP definition, frequency, and risk factors. Individual items of the CPMS were also described. Descriptive statistics were stratified based on participants’ profiles—that is, groups formed by those reporting suffering from CP (yes/no) or those reporting being a health care professional (yes/no). A multiple linear regression model was used to identify participant characteristics that were associated with knowledge, beliefs, and attitudes toward people suffering from CP as measured by the CPMS first subscale score (dependent variable). All independent variables were treated simultaneously in the model. Results are presented as crude and adjusted β, standard errors, and their respective *P* values. All statistical analyses were performed using IBM SPSS Statistics version 22 (IBM Corp, Armonk, NY) and SAS version 9.3 (SAS Institute, Cary, NC).

## Results

### Participant characteristics

A total of 1958 participants completed the web-based questionnaire (Table 1). Participants’ ages ranged from 18 to 83 years (mean ± SD: 49.1 ± 13.1) and most were women (78.1%). The survey reached participants with various socioeconomic statuses from the 17 administrative regions of the province. A majority of participants reported pain for ≥3 months (70.9%), who were considered to be CP sufferers. Physicians, nurses, physiotherapists, psychologists, or pharmacists represented 14.4% of the study sample. The proportion of missing data did not reach the cutoff (5%–15%) for which they are known to be problematic.^53^

**Table 1.** Study population’s characteristics.

Characteristics^a^ *n* = 1958	No. (%) of participants^b^
Age (years), mean ± SD	49.12 ± 13.09
Min	18
Max	83
Sex	
Female	1465 (78.09)
Male	411 (21.91)
Country of birth	
Canada	1747 (93.62)
Other	119 (6.38)
Employment status	
Full-time job	915 (48.88)
Part-time job	158 (8.44)
Unemployed	799 (42.68)
Living conditions	
Living alone	488 (26.01)
Living with spouse/common-law partner	1276 (68.02)
Other^c^	112 (5.97)
Annual family income (CAD)	
Less than 20 000	250 (13.77)
20 000–39 999	320 (17.63)
40 000–59 999	345 (19.01)
60 000–79 999	304 (16.75)
80 000–99 999	226 (12.45)
100 000 and over	370 (20.39)
Completed education level	
Elementary	21 (1.12)
High school	314 (16.70)
Diploma in vocational studies	248 (13.19)
College/CÉGEP	406 (21.60)
University–Undergraduate studies	528 (28.09)
University–Graduate studies	363 (19.31)
Region of residence	
Nonremote regions	1311 (70.98)
Residents of remote resource regions^d^	536 (29.02)
Suffering from CP (pain for ≥3 months)	
Yes	1329 (70.92)
No	545 (29.08)
Knowing someone who suffers from CP	
Yes	1574 (83.72)
No	306 (16.28)
Had worked or studied in the field of health^e^	
Yes	759 (40.33)
No	1123 (59.67)
Had worked with patients claiming CP disability benefits (e.g., CSST, SAAQ, insurances)	
Yes	252 (13.70)
No	1587 (86.30)
Health care professionals^f^	
Yes	269 (14.42)
No	1597 (85.58)

^a^Proportion of missing data across presented variable ranges between 3.9% and 7.3%.

^b^Unless stated otherwise.

^c^Including living with parents, cotenant(s), or in a seniors’ residence.

^d^Remote resource regions as defined by Revenu Quebec (i.e., the provincial revenue agency): Bas-Saint-Laurent (region 01), Saguenay–Lac-Saint-Jean (region 02), Abitibi-Témiscamingue (region 08), Côte-Nord (region 09), Nord-du-Québec (region 10), Gaspésie–Îles-de-la-Madeleine (region 11). Nonremote regions are near a major urban center.

^e^Including health care professionals (physician, nurse, physiotherapist, psychologist, or pharmacist) but also fields such as health administration, health promotion, education, kinesiology, nutrition, paramedical, beneficiary attendance, laboratory technology, research, etc.

^f^Physician, nurse, physiotherapist, psychologist, or pharmacist.

CAD = Canadian dollars; CÉGEP = Collège d'enseignement général et professionnel; CP: chronic pain; CSST = Commission de la santé et de la sécurité au travail; SAAQ = Société de l'assurance automobile du Québec.

### Description of knowledge, beliefs, and attitudes

Results of all true or false questions about specific aspects of CP definition, frequency, and risk factors are depicted in Table 2 (for each item, the proportion of participants who had the incorrect answer is presented in order to better represent knowledge gaps). Among the respondents without CP, with CP, or health care professionals, the two most common knowledge gaps were the facts that the risk of developing CP is increased after undergoing surgery and that CP affects approximately one in five adults.

**Table 2.** Knowledge about specific aspects of CP definition, frequency, and risk factors.

True or false statements	Proportion of participants who had the incorrect answer (%)			
	Participants without CP *n* = 545	Participants suffering from CP *n* = 1329	Heath care professionals *n* = 269	Whole sample *n* = 1958
The risk of developing CP is increased after undergoing surgery (*true*)	53.96	56.18	43.87	55.66
In the province of Quebec, CP affects approximately one in five adults (*true*)	48.62	41.09	27.14	43.52
With the aging population, a considerable increase in the number of CP cases is expected in the years to come (*true*)	29.13	28.44	18.35	28.74
CP is a disease, just like other chronic diseases such as diabetes (*true*)	28.49	24.83	19.40	26.27
Children (including newborns) experience less pain than adults (*false*)	27.26	28.32	14.50	28.81
CP can be defined as having pain daily, or repeatedly, for at least 3 months (*true*)	19,85	14.42	12.64	15.98
Fibromyalgia is a form of mental illness (*false*)	11.23	9.21	4.09	9.95
CP is mainly “between the ears,” more psychological than physical (*false*)	6.46	3.25	5.20	4.31

CP = chronic pain.

A minority of participants (10.30%) agreed that health care professionals such as physicians, pharmacists, and nurses are well trained in CP treatment. This proportion was consistently low among the different subgroups (participants without CP: 10.97%; participants suffering from CP: 10.03%; health care professionals themselves: 7.12%).

In Table 3 the most common negative beliefs as measured by individual items of the CPMS are presented. The two most frequent negative beliefs among all subgroups were that people suffering from CP become dependent on their medications as do people with drug addiction and that consulting a psychologist is useless unless the person with CP is depressed.

**Table 3.** Negative beliefs reported by study participants.

Individual items of the CPMS	Proportion of participants who agreed with these statements (%)			
	Participants without CP *n* = 545	Participants suffering from CP *n* = 1329	Heath care professionals *n* = 269	Whole sample *n* = 1958
Become dependent on their medications as do drug addicts (item #8)	23.07	14.30	16.04	16.70
Consulting a psychologist is useless unless the person with CP is depressed (item #20)	14.44	17.97	7.49	16.89
There is not much to do to improve CP (item #21)	5.95	12.33	6.34	10.46
People who suffer from CP complain about their pain but continue their activities (e.g., sports, snowmobile). Their pain should not be that bad! (item #7)	9.02	6.97	7.49	7.87

CPSM = Chronic Pain Myth Scale; CP = chronic pain.

### Predictors of knowledge, beliefs, and attitudes toward people suffering from CP

Simple and multiple linear regression analyses aimed at identifying participant characteristics that were associated with knowledge, beliefs, and attitudes toward people suffering from CP as measured by the CPMS subscale score are presented in Table 4. The analysis revealed that independent of other characteristics included in the model, being a woman (β = 1.105, *P* < 0.0001), being born in Canada (β = 1.263, *P* = 0.0085), being unemployed (β = 0.815, *P* = 0.0046), suffering from CP (β = 1.767, *P* < 0.0001), and being trained as a physician, nurse, physiotherapist, psychologist, or pharmacist (β = 0.806, *P* = 0.0206) were predictors of better knowledge and more positive beliefs and attitudes toward people suffering from CP. Older age (β = −0.022, *P* = 0.0204), residing in a remote resource region of the province (β = −0.553, *P* = 0.0180), and having worked or studied in the field of health independently of being a health care professional (β = −0.753, *P* = 0.0026) were associated with poorer knowledge and more negative beliefs and attitudes.

**Table 4.** Participants’ characteristics associated with knowledge, beliefs, and attitudes toward people suffering from CP as measured by the CPMS.

Characteristics	Simple linear regression models			Multiple linear regression model^a^		
	Crude β	SE	*P* value	Adjusted β	SE	*P* value
Age	0.009	0.008	0.2352	−0.022	0.010	**0.0204**
Sex (vs. males) Females	1.182	0.244	<0.0001	1.105	0.256	**<0.0001**
Country of birth (vs. other) Canada	1.027	0.416	0.0138	1.263	0.479	**0.0085**
Employment status (vs. full-time job)						
Part-time job	0.065	0.372	0.8615	0.071	0.402	0.8600
Unemployed	1.437	0.211	<0.0001	0.815	0.287	**0.0046**
Living conditions (vs. alone)						
Living with spouse/common-law partner	−0.199	0.233	0.3937	0.330	0.274	0.2289
Other	−0.137	0.458	0.7653	0.170	0.492	0.7291
Annual family income (vs. 60 000–79 999 CAD^b^)						
Less than 20 000	0.160	0.373	0.0019	0.656	0.443	0.1387
20 000–39 999	0.207	0.349	0.5526	−0.190	0.375	0.6130
40 000–59 999	0.049	0.343	0.8867	−0.118	0.355	0.7396
80 000–99 999	−0.366	0.381	0.3369	−0.151	0.385	0.6949
100 000 and over	−0.394	0.336	0.2417	−0.027	0.355	0.9386
Completed education level (vs. elementary or high school)						
Diploma in vocational studies	0.354	0.367	0.3354	0.319	0.387	0.4101
College/CÉGEP	−0.340	0.326	0.2967	0.091	0.353	0.7977
University	−0.859	0.282	0.0024	0.043	0.330	0.8963
Residents of remote resource regions (vs. nonremote regions)	−0.591	0.227	0.0092	−0.553	0.234	**0.0180**
Suffering from CP (yes vs. no)	2.100	0.218	<0.0001	1.767	0.252	**<0.0001**
Knowing someone who suffers from CP (yes vs. no)	0.625	0.274	0.0225	0.359	0.285	0.2083
Had worked or studied in the field of health (yes vs. no)	−0.684	0.206	0.0009	−0.753	0.249	**0.0026**
Had worked with patients claiming CP disability benefits (yes vs. no)	−0.905	0.297	0.0024	−0.519	0.326	0.1118
Health care professionals (yes vs. no)	−0.182	0.291	0.5326	0.806	0.348	**0.0206**

^a^Multicollinearity: For all variables included in the multiple model, variance inflation factors were below <3.

^b^Category which represents the 2014 median total income by family in the province of Quebec.^54^

^c^Bold values in the table represent predictors with P values < 0.05.

CP = chronic pain; CPSM = Chronic Pain Myth Scale; CAD = Canadian dollar; CÉGEP = Collège d'enseignement général et professionnel.

## Discussion

The present web-based study conducted among various subgroups of the Quebec population was aimed at identifying the main knowledge gaps and negative beliefs/attitudes maintained regarding CP, especially toward people suffering from this condition.

This study suggests a lack of awareness regarding the frequency of CP and other elements surrounding its recognition, in the community, among participants who suffer from this condition, and among health care professionals. The most common knowledge gap was regarding the statement stipulating that the risk of developing CP is increased after undergoing surgery; that is, 55.66% of respondents reported this statement to be false or did not know the answer (43.87% of health care professionals). Yet, chronic postsurgical pain is a common complication of surgery that has been discussed for the last 20 years.^55,56^ Although the need for better education of health care professionals and the general public was highlighted,^55^ the results of our study unfortunately underline that there is still some way to go before the problem is more widely known in the general population and among health care professionals. On the other hand, awareness and education initiatives should be well designed and framed to reduce the risk of exacerbating fear in patients anticipating surgery.

Our results reflect a negative opinion about health care professionals’ training in CP treatment. This contrasts with Canada’s general population reporting high levels of trust in health care professionals and rating their overall knowledge as good.^57^ Patients’ trust in physicians, which is partly based on perceived competence and personal experience, can affect satisfaction and adherence to treatment.^58^ It was also demonstrated that lack of trust in health care professionals could lead patients suffering from chronic conditions to be more inclined to ask for a second opinion (e.g., doctor shopping) or use complementary and alternative medicines,^59^ which may in turn result in a greater societal cost of illness. The present study thus provides an additional argument to enhance resource allocation in prelicensure pain curricula and continuing education activities about CP and its treatment. In fact, knowledge gaps about CP and its treatment among health care professionals constitute an important barrier to adequate management of this condition.^11^ Limited knowledge of pain and negative attitudes toward CP patients have also been shown to limit the use of clinical practice guidelines.^60^ More efficient dissemination of recent advances in the field of health care professionals’ education among the general public is also desirable.

Negative beliefs and attitudes regarding people who suffer from CP and treatment of CP were present in a small but nonnegligible proportion of study participants. For example, 23.07% of participants without CP agreed that people suffering from CP become dependent on their medications as do drug addicts (14.30% of participants with CP, 16.04% of health care professionals). This result is not surprising given that the negative attention toward opioids contributes to stigmatization of people suffering from CP.^61^ Stigma reduction activities targeting the general public should address this problem, especially because perceived social and family support are important factors associated with clinical outcomes among CP patients.^62,63^

Although psychologists are identified by the International Association for the Study of Pain as one of the classes of professionals who should be involved in multidisciplinary CP management^51^ and evidence of effectiveness of psychological interventions is well established,^64^ 17.97% of participants with CP agreed that consulting a psychologist is useless unless the person with CP is depressed. It is not clear whether these respondents were referring to their experience with such health care professionals or made this assumption, but it has been previously reported that some patients are skeptical about the usefulness and benefits of psychological treatments for pain.^65^ This emphasizes another important topic that should be covered in future awareness and educational campaigns.

The need to better understand determinants of the stigma associated with CP was recently underlined.^20^ Our results suggest some differences in knowledge, beliefs, and attitudes toward people suffering from CP across different sociodemographic, occupational, and health profiles. Studying respondents’ characteristics associated with such perceptions will help better target awareness and education activities. For example, older individuals, men, newcomers to Canada, people without CP, residents of remote regions, and students/workers in paramedical/biomedical disciplines should be targeted. In line with our results, a previous study about attitudes of the general public toward morphine also showed that older individuals, men, and residents of rural areas had more negative beliefs.^42^ Contrary to what was expected, being unemployed (as opposed to a having a full-time job) was a predictor of better knowledge and more positive beliefs and attitudes toward people suffering from CP, even after controlling for other sociodemographic variables and presence of CP. A possible explanation could be that nonworking participants are more likely to have chronic disease disability benefits, a variable not measured in the present study. Previous studies showed that people with disability had more positive attitudes toward people with disability.^66^

In the past years, heightened awareness and increased education about CP has been an integral part of many Canadian pain-related initiatives, including the annual Pain Awareness Week (first week of November put forward by provincial and national organizations such as the Quebec Association for Chronic Pain^45^ and the Canadian Pain Coalition^44^) and knowledge translation initiatives such as the Programme ACCORD.^46^ The benefits of population-based campaigns such as the Canadian Back Pain Mass Media Campaign^47^ have also been demonstrated.^40,47,67^ It should also be noted that considerable effort has been invested in enhancing health care professionals’ prelicensure education in pain management.^48^ The results of the present study, however, underline the importance of continuing these efforts and the need for more education programs, awareness campaigns, and stigma reduction activities about CP. We can ask ourselves whether knowledge translation activities carried out to date have been effective and what we can do to improve them. The findings of the present study may be useful in this regard and be used to better design and tailor such activities. Ultimately, greater knowledge and recognition of CP in the population could facilitate its management. In the context of other chronic health conditions, improved public awareness contributed to more rapid diagnosis and treatment.^68^

### Strengths and limitations

Strengths of the present study include the careful development and pretesting of the web-based questionnaire, the minimization of errors in computerizing the data by the use of a commercial survey software, and the utilization of a multiple regression model reducing the possibility of confounding bias for the identification of the predictors of knowledge, beliefs, and attitudes toward people suffering from CP. The possibility of type II error was also minimized due to the substantial sample size (according to published recommendations, sample size divided by two represents the number of variables that can be included in a multiple linear model^69^). However, the cross-sectional nature of the present study limits assessment of causal relationships between participants’ characteristics and lower knowledge/more negative beliefs and attitudes toward people suffering from CP. Moreover, we cannot exclude the possibility of a social desirability bias in participants’ responses. In the development process of our questionnaire, we avoided distinguishing items as measuring either knowledge, beliefs, or attitudes as suggested by the authors of the Knowledge and Attitudes Survey Regarding Pain.^70^ However, in the interpretation of our findings, we have to keep in mind that knowledge and beliefs are not always the same (e.g., knowing something does not mean that you believe it).

Despite the impossibility of calculating a participation rate and reaching people without Internet access, participants had various socioeconomic profiles and came from different geographic areas of the province, which increases the ability to generalize our findings. There is thus a reason to believe that knowledge, beliefs, and attitudes toward people suffering from CP maintained by Quebec residents are unlikely to be isolated to our province or country. It should be noted that patients suffering from CP, people interested in pain, and women appeared more inclined to take part in our study, which could impact the results. A way to circumvent this limit was to stratify descriptive statistics based on participants’ profiles and to include all participant characteristics in the multiple regression model aimed at identifying predictors of knowledge, beliefs, and attitudes toward people suffering from CP.

### Conclusion

On the basis of the results of this research, it can be concluded that the prevalence of CP is not well known and that negative attitudes continue to be held in different subgroups of the Quebec population. An improved recognition of CP as well as heightened awareness levels and increased education for health care professionals, patients, and the general public is needed.
